# Editorial

**Published:** 2013-05-09

**Authors:** Shibal Bhartiya, Tarek Shaarawy, Tanuj Dada

‘If you have knowledge, let others light their candles with it.’

—Wiston Churchill

As clinicians and academicians, our fundamental goal is to share our experiences and knowledge with our students and colleagues. It is a pleasant and rewarding responsibility and the editorial team of JOCGP really cherishes it.

We take this opportunity to thank our contributors for sharing their ideas and experiences with the fraternity.

The boom in the diagnostic technology for early detection of glaucoma has been astounding. Danekal et al threw insight on role of pattern electroretinogram (PERG) in detection of early glaucoma. They found that the P50 and N95 amplitude of patients with primary open angle glaucoma (POAG) and glaucoma suspect were significantly reduced and N95 amplitude was the better indicator for diagnosis of POAG when used individually.

In another study, Asrani et al carried out noninvasive imaging of Schlemm’s canal and trabecular meshwork in vivo with Fourier domain optical coherence tomography (FDOCT) to visualize ocular angular structures crucial for glaucoma management. They report that the selection of the OCT operating wavelength and exact location of the scan across different meridia minimally affects the appearance of the ocular anatomy.

Health provisions vary around the globe. Some sectors do not have access to such sophisticated and objective imaging devices so clinical assessment helps to detect the disease. Bhartiya and Shaarawy evaluated the usefulness of the Van Herick technique in ruling out narrow angles in glaucoma patients in Southern Egypt. They found that even seemingly deep limbal anterior chamber could have an occludable angle on gonioscopy. They recommend that all ophthalmologists must make the final assessment with a gonioscope.

Rajkumari et al bring forward the scenario of North-eastern region of India, which continues to suffer from limited resources, added upon by Mongoloid racial similarity and poor cataract surgery rate. This has significantly contributed to the increased incidence of advanced cataract like phacomorphic glaucoma. They evaluate the role of manual small incision cataract surgery in controlling the IOP and achieving good visual acuity with minimal complications.

A review by Alon looks at recently published studies on the mechanisms and clinical outcome of selective laser trabeculoplasty (SLT) and attempts to address issues pertinent to SLT in the clinical practice. The key to understanding any disease entity is to understand the basic biochemical concepts. A review by Ahmed et al throws light on the new developments which form the biochemical basis of glaucomatous neural degeneration. The role of excitatory amino acids, caspases, protein kinases, oxygen free radicals, nitric oxide, TNF-alpha, neurotrophins and metalloproteins in causation of neurodegeneration has been highlighted.

Faiq et al discussed the various clinical, biochemical and genetic aspects of primary congenital glaucoma. They emphasized on the fact that the etiology of this glaucoma subtype does not lie merely in a single gene or genetic factor. Gene-gene interactions, ocular embryology, ophthalmic metabolism and systemic oxidative status need to be studied to unravel the mystery surrounding this disorder.

Many patients have coexisting cataract and glaucoma. IOL selection plays a vital role in optimizing visual outcomes and minimizing complications in such patients. Ichhpujani et al collated the evidence regarding the use of premium intraocular lenses in patients with glaucoma.

Case reports have often been disregarded in medical literature but there is no denying the fact that they provide important information about individuals which is often not cited in larger studies. Chakraborty and Spaeth report an interesting case of overlapping syndrome with advanced pigmentary glaucoma and Marfan syndrome.

As always, the editorial team looks forward to hearing from you.

Best wishes

**Shibal Bhartiya**

**Tarek Shaarawy**

**Tanuj Dada**

